# Motor Cortex and Hippocampus Display Decreased Heme Oxygenase Activity 2 Weeks After Ventricular Fibrillation Cardiac Arrest in Rats

**DOI:** 10.3389/fmed.2020.00513

**Published:** 2020-09-10

**Authors:** Alexandra-Maria Warenits, Jasmin Hatami, Andrea Müllebner, Florian Ettl, Ursula Teubenbacher, Ingrid Anna Maria Magnet, Barbara Bauder, Andreas Janata, Ingrid Miller, Rudolf Moldzio, Anne-Margarethe Kramer, Fritz Sterz, Michael Holzer, Sandra Högler, Wolfgang Weihs, Johanna Catharina Duvigneau

**Affiliations:** ^1^Department of Emergency Medicine, Medical University of Vienna, Vienna, Austria; ^2^Institute for Medical Biochemistry, University of Veterinary Medicine Vienna, Vienna, Austria; ^3^Ludwig Boltzmann Institute for Experimental and Clinical Traumatology, Vienna, Austria; ^4^Institute of Pathology, University of Veterinary Medicine Vienna, Vienna, Austria; ^5^Center for Biomedical Research, Medical University of Vienna, Vienna, Austria; ^6^Unit of Laboratory Animal Pathology, University of Veterinary Medicine Vienna, Vienna, Austria

**Keywords:** cardiac arrest, global cerebral ischemia, reperfusion injury, heme degradation pathway, brain regions, neurodegeneration, biliverdin reductase, enzyme activity

## Abstract

Heme oxygenase (HO) and biliverdin reductase (BVR) activities are important for neuronal function and redox homeostasis. Resuscitation from cardiac arrest (CA) frequently results in neuronal injury and delayed neurodegeneration that typically affect vulnerable brain regions, primarily hippocampus (Hc) and motor cortex (mC), but occasionally also striatum and cerebellum. We questioned whether these delayed effects are associated with changes of the HO/BVR system. We therefore analyzed the activities of HO and BVR in the brain regions Hc, mC, striatum and cerebellum of rats subjected to ventricular fibrillation CA (6 min or 8 min) after 2 weeks following resuscitation, or sham operation. From all investigated regions, only Hc and mC showed significantly decreased HO activities, while BVR activity was not affected. In order to find an explanation for the changed HO activity, we analyzed protein abundance and mRNA expression levels of HO-1, the inducible, and HO-2, the constitutively expressed isoform, in the affected regions. In both regions we found a tendency for a decreased immunoreactivity of HO-2 using immunoblots and immunohistochemistry. Additionally, we investigated the histological appearance and the expression of markers indicative for activation of microglia [*tumor necrosis factor receptor type I* (TNFR1) mRNA and immunoreactivity for ionized calcium-binding adapter molecule 1 (Iba1])], and activation of astrocytes [immunoreactivity for glial fibrillary acidic protein (GFAP)] in Hc and mC. Morphological changes were detected only in Hc displaying loss of neurons in the cornu ammonis 1 (*CA1*) region, which was most pronounced in the 8 min CA group. In this region also markers indicating inflammation and activation of pro-death pathways (expression of HO-1 and TNFR1 mRNA, as well as Iba1 and GFAP immunoreactivity) were upregulated. Since HO products are relevant for maintaining neuronal function, our data suggest that neurodegenerative processes following CA may be associated with a decreased capacity to convert heme into HO products in particularly vulnerable brain regions.

## Introduction

Neurologic outcome in patients resuscitated from cardiac arrest (CA) remains poor, despite improvements in advanced life support and post-resuscitation care. Brain injury starts during the initial CA (no-flow time), continues during resuscitation (low-flow time) and culminates after re-oxygenation with the return of spontaneous circulation (ROSC). These pathophysiological events prepare the base of the post-cardiac arrest syndrome (1, 2) and lead to neurologic and motor deficits (3), which were shown to persist for a long time (4, 5). Using animal models, like others we have previously shown that CA induced global ischemia causes delayed neurodegeneration and neuronal dysfunction, which is associated with decreased cognitive capabilities (6–9). An experimental study using rats revealed that cognitive deficits in response to global ischemia continue to develop and were most severe after 6 months when rats are in their middle age (10). The same group also showed that cognitive deficits produced by ischemia are severe in middle aged rats, suggesting that repair mechanisms decline with age (11).

Multiple mechanisms are initiated immediately after ROSC involving an imbalanced redox homeostasis (12), increased cell stress, initiation of inflammation and cell death signaling (13–15) followed by death of vulnerable neurons. The most frequent structural changes occur in the hippocampus (Hc), consisting of loss of pyramidal neurons in the cornu ammonis 1 (*CA1*) region (16), which are associated with behavioral alterations (17).

The heme oxygenase (HO) system supports neuronal function and contributes to the oxidative defense (18, 19). HO degrades heme via oxidation yielding carbon monoxide (CO), free iron, and biliverdin (BV), which is afterwards converted to bilirubin (BR) (19). There are two catalytically active isoforms of HO in mammals, HO-1 and HO-2. In neuronal tissue nearly all of the HO-activity is ascribable to the constitutive HO isoform HO-2, and contribution of HO-1 is almost absent in physiological conditions (20). Functional HO is required for modulation of the synaptic activity, for memory consolidation, and maintenance of microperfusion, which operates predominantly via the heme degradation product CO (21–23).

Apart from the exclusive role of HO-2 for maintaining homeostasis and function in neuronal tissues, upregulation of the inducible isoform of HO-1, synonym with heat shock protein 32 is observed in response to acute cell stress, such as hypoxia and ischemia (24). Within several hours cerebral ischemia leads to the induction of HO-1, initially in astrocytes, which is subsequently extended on neuronal cells (25–27). Additionally to the HO-1 induction, also up-regulation of HO-2 protein has occasionally been described in response to cerebral ischemia (28). Experimental models inhibiting HO or overexpressing HO prior to an ischemic insult documented the neuroprotective effects of HO, particularly that of HO-2 (29). HO mediated cytoprotection can be mimicked by application of the HO reaction products CO (30) or BV/BR (31, 32).

Given the particular role of HO in preserving neuronal morphology and function, we questioned whether delayed neurodegeneration caused by resuscitated cardiac arrest is associated with changes of HO activity. Most studies delimitate their investigation to the expression of mRNA or protein level of either HO-1 or HO-2, or both HO isoforms in a few cases. Several studies show an acutely increased mRNA and/or protein expression of the HO-1 isoform in response to global ischemia (28, 29). However, HO activity of brain regions following CA has not been investigated yet. Instead, HO activity is extrapolated from the observed expression levels of HO-1 and/or HO-2 mRNA or protein, although it is known that both enzymes, HO-1 and HO-2, may be subjected to posttranslational modifications, influencing their catalytic activity (33, 34). Therefore, the capacity to degrade heme may be different from what mRNA or protein levels suggest.

Thus, our study aimed at understanding whether HO activity and expression do indeed correlate in vulnerable brain regions, as is generally assumed. To test our hypothesis we applied an experimental ventricular fibrillation model of 6 or 8 min CA, followed by cardiopulmonary resuscitation (CPR) using rats (7). After 2 weeks, we analyzed in Hc, motor cortex (mC), striatum and cerebellum the activity and expression levels of enzymes of the heme degradation pathway. To understand whether changes of the HO system are associated with neurodegenerative processes, we further analyzed the affected regions for relevant markers indicating gliosis and activation of inflammatory cell death pathway processes.

## Materials and Methods

### Animals and Experimental Protocol

The experimental protocol was approved by the Institutional Animal Care and Use Committee of the Medical University of Vienna and the Austrian Ministry of Science, Research and Economy (GZ.: 66.009/0064-II/3b/2011). The experiments were conducted in compliance with EU regulations for animal experimentation (Directive 2010/63/EU of the European Parliament and of the Council) and followed the ARRIVE guidelines (35).

A total of 32 adult male Sprague-Dawley rats, 389 ± 56 g body weight (BW), 10 weeks of age (Himberg, Austria) were randomly allocated into the two resuscitation groups of 6 and 8 min CA (6 min CA, *n* = 10; 8 min CA, *n* = 12) and a sham operated group (sham, *n* = 10). In the 8 min CA group, more animals were allocated, because we expected a higher loss of animals due to longer CA times. In the sham group the same surgical procedures were performed, without the induction of ventricular fibrillation and consequent CA.

The experimental resuscitation model was described in detail earlier (7). Briefly, sedation of the animals was induced via the administration of sevoflurane 6% in FiO_2_ 1.0 for 4 min in a box. The rats were endotracheally intubated with an adapted venous cannula (14GA Venflon BD Luer-Lok, Helsingborg, Sweden) and mechanically ventilated, volume-controlled with 65/min, 7 mL/kg BW and 0.3 FiO_2_ (Havard Inspira advanced safety ventilator, volume controlled, MA1 55-7058, Holliston, MA, USA). Buprenorphine (50 μg/kg BW) was given subcutaneously (s.c.) after intubation and sevoflurane 3.5% was further administered via the ventilator for continuous anesthesia during surgery. Rectal and esophageal temperature was kept stable at 37 ± 0.2°C with a heated operating table. The animals were catheterized via surgical cut-down into the left femoral artery and vein (Argyle Polyurethane Umbilical Vessel Catheter; 2.5 Fr, Convidien, Mansfield, MA, USA) for arterial blood sampling, measurement of mean arterial pressure (MAP) and venous drug administration. A human neonatal pacing catheter (Vygon GmbH & Co Bi-Pacing-ball 3 Fr, Aachen, Germany) was inserted in the cranially ligated right jugular vein, with the tip ending in the inferior vena cava for inducing ventricular fibrillation cardiac arrest.

After successful surgery, a baseline arterial blood gas analysis was performed, and sevoflurane sedation was stopped 1.5 min before the induction of ventricular fibrillation and the end of mechanical ventilation. After untreated CA (6 min CA, or 8 min CA), bicarbonate (1 mmol/kg BW), unfractionated heparin (100 I.U.), and epinephrine were given (20 μg/kg BW) intravenously (i.v.) 60 s before the initiation of resuscitation. Resuscitation was started with mechanical chest compressions (200/min) delivered with a pneumatic chest compression device (Streubel Automation, Grampersdorf, Germany) and mechanical ventilation (20/min, 7 mL/kg BW, 1.0 FiO_2_). The animals were defibrillated (2 times each with 5 Joule, biphasic; repeated every 2 min if ventricular fibrillation was present) and received epinephrine (10 μg/kg BW) i.v. 60 s after start of CPR and repeated every 2 min to achieve ROSC.

After ROSC, the ventilation settings were adapted (65/min, 7 mL/kg BW and 0.5 FiO_2_) and an arterial blood gas sample was taken 5 min after ROSC. The catheters were removed, vessels ligated, and skin incisions sutured using aseptic techniques. After successful weaning from mechanical ventilation, the unconscious rats were extubated, received oxygen via a nose cone mask and buprenorphine (24 μg/kg BW) s.c. as long as pain or distress was present. Further information regarding clinical data (mean arterial pressure, blood gas analyses prior and after CA and ROSC) is given as Table S1.

**Figure 1:**
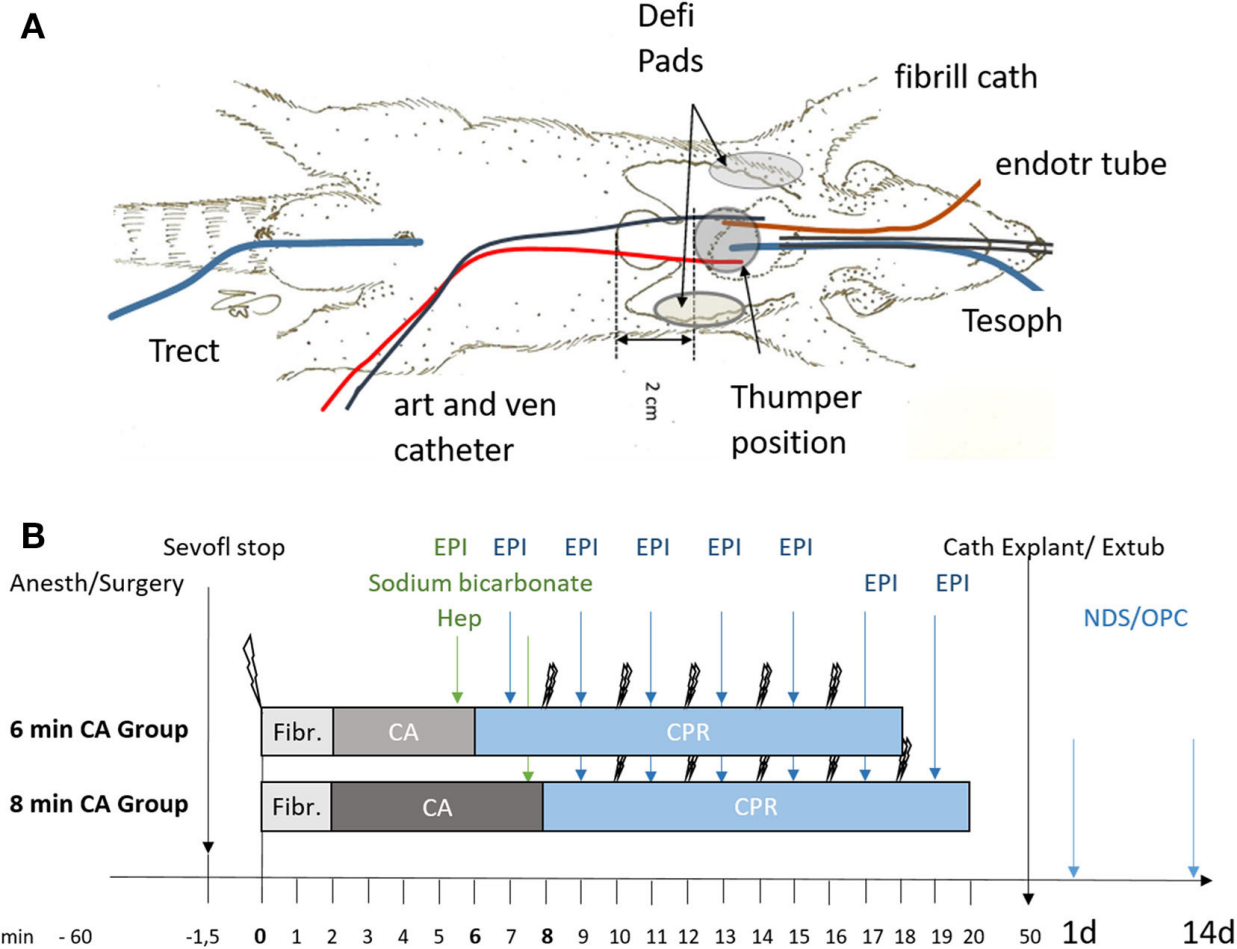
Schematic representation of setup and timeline of the experiments. **(A)** Instrumentation of animals using an endotracheal tube (endotr tube), an arterial and venous catheter in the femoral artery and vein (art and ven catheter), an esophageal temperature probe (Tesoph), and a rectal temperature probe (Trect). Fibrillation catheter for inducing ventricular fibrillation (fibrill cath). For resuscitation defibrillation pads (Defi Pads) were placed as indicated and a thumper was positioned 2 cm cranial of the xiphoid process. **(B)** Animals were randomly allocated to sham (not shown) or the 6 or 8 min cardiac arrest (CA) group following anesthesia and surgery (Anesth/Surgery). Sevoflurane was stopped (Sevofl stop) and CA was induced by ventricular fibrillation (single lightning symbol; Fibr.). Heparin (Hep), epinephrine (EPI) and sodium bicarbonate were added prior to cardiopulmonary resuscitation (CPR) attempted by 2 consecutive defibrillation steps (double lightning bolts), repeated every 2 min of CPR and followed immediately by epinephrine (EPI) supplementation every 2 min of CPR. After successful resuscitation with maximal 5 defibrillation attempts catheters were explanted (Cath Explant) and animals extubated (Extub). Overall performance category score (OPC) and neurological deficit score (NDS) were determined in all surviving animals at day 1 and day 14 prior to sacrifice.

The rats were evaluated daily and neurologic status was assessed on day 1 and day 14 after ROSC by an investigator blinded to the study group, using neurological deficit score (NDS; NDS 0% = normal, NDS = 100% dead) (36) and overall performance category score (OPC; OPC 1 = normal, 2 = moderate disability, 3 = severe disability, 4 = comatose, 5 = dead (37). The neurological scoring system of NDS and OPC is described in the Tables S2, S3. A schematic overview of the experimental setup (a) and the study timeline (b) is shown in Figure 1.

### Sampling

**Figure 2:**
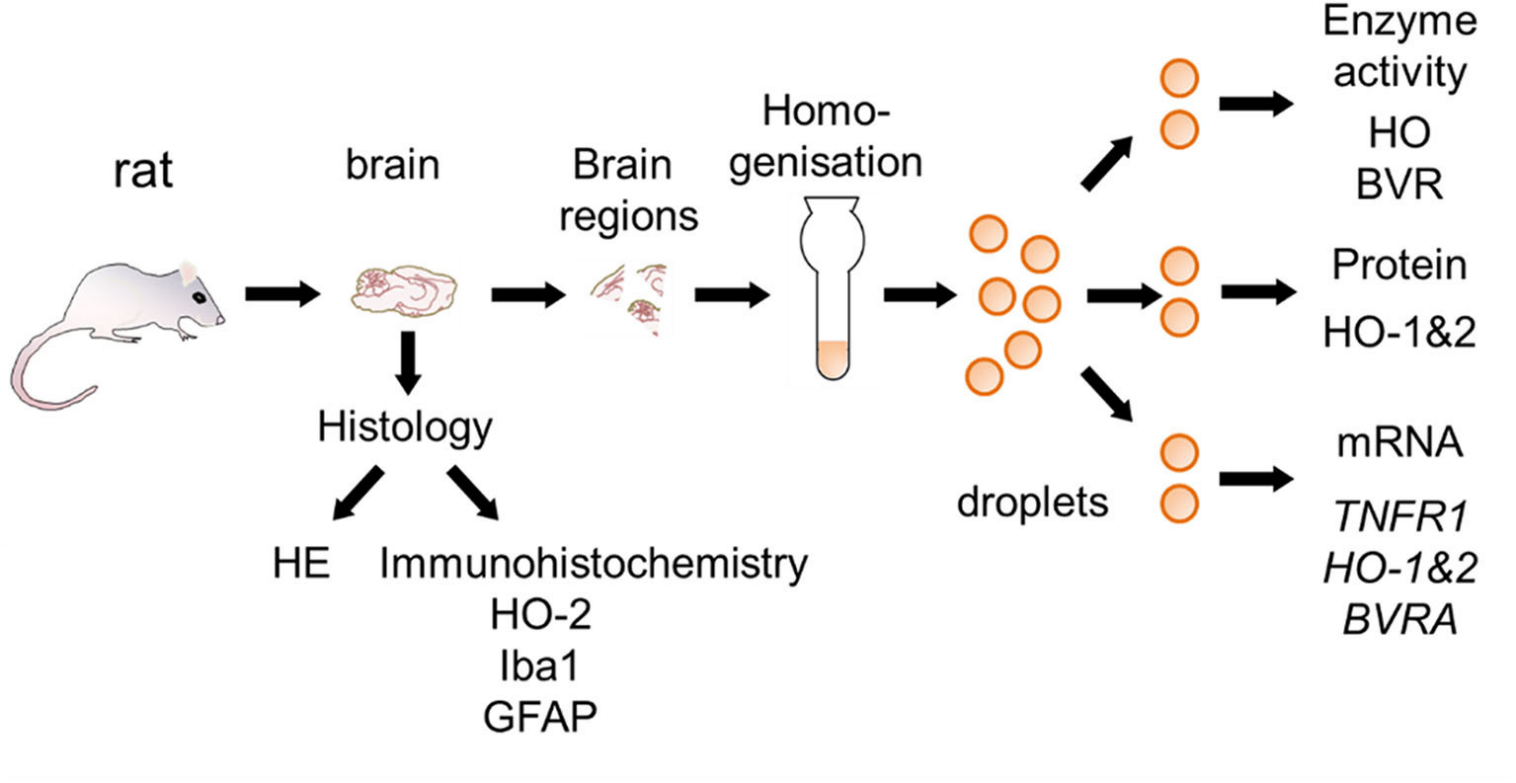
Schematic overview for the preparation of brain sections used for the different analysis. Brain samples were taken from rats surviving 2 weeks following CA and processed as described in the Materials and Methods section. Half of the brain was fixed and used for histological examination [hematoxylin and eosin staining (HE), immunohistochemistry for heme oxygenase (HO)-2, ionized calcium-binding adapter molecule 1 (Iba-1), and glial fibrillary acidic protein (GFAP)]. Different regions were cut from the other half and immediately snap-frozen. In order to prevent biases frozen tissue pieces were homogenized and distributed into droplets prior to analysis of activity of HO and BVR, protein expression of HO-1 and HO-2, and gene expression [*Tumor necrosis factor receptor 1* (TNFR1), HO-1, HO-2, and *biliverdin reductase A* (BVRA)]. Analyses were performed in accordance to the procedures described in Materials and Methods section.

At day 14 rats were euthanized with a sevoflurane and buprenorphine overdose and perfused with saline (0.9% NaCl). After perfusion, brains were removed from the skull and cut into hemispheres. One half was fixed in 7.5% neutral buffered formaldehyde solution for histological examination, and regions of the other hemisphere (mC, Hc, striatum, and cerebellum) were sampled for gene expression analysis and enzyme assays. For this purpose, coronary sections at Bregma 1.7, −1.4, and −5.2 (38) were cut. The mC was taken from the rostral section, striatum from the next section and Hc from the third section. Cerebellar peduncles were cut to remove the cerebellum from the brain stem. The entire sampling process was done on a chilled marble plate and lasted not longer than 30 min. Brain regions were snap frozen in liquid nitrogen and stored at −80°C until use. The entire procedure with analyses performed is shown in Figure 2.

### Preparation of Tissue Homogenates for Enzyme Assays and Expression Analysis

Homogenates of entire brain regions were prepared (Figure 2) instead of using only parts/pieces, in order to avoid biases due to potential tissue inhomogeneity. These homogenates were used for the determination of the HO and BVR activity and for the protein and gene expression analyses. Frozen tissue was homogenized in 1:20 (w/v) Tris-buffer containing 300 mM sucrose, 20 mM Tris, and 2 mM EDTA at pH 7.4 using a Potter-Elvehjem with PTFE pestle on ice. The homogenates were distributed in 30 μL portions directly into liquid nitrogen. The formed frozen droplets were stored at −80°C until being used.

### Determination of the Activity of HO Enzyme by an Optimized Photometric Enzyme-Coupled Assay

The determination of enzyme activities (HO and BVR) was performed as previously described (39) with the following modifications. Two droplets (corresponding to approximately 1 mg of protein) were added to a reaction mixture containing 500 nmol NADPH (Sigma) in a total volume of 150 μL assay buffer (100 mM potassium phosphate buffer; 1 mM EDTA; pH 7.4), supplemented with 20 nmol of hemin (for determination of HO activity), or with 200 nmol BV (for determination of BVR) and 250 nmol of NADPH. The residual homogenate was used to determine the protein concentration using a Coomassie Brilliant Blue binding assay (Bradford), as described elsewhere (40). The mixture was incubated under constant agitation in darkness for 30 min at 37°C. The reaction was stopped by transferring the samples onto ice. BR was extracted into benzene as described previously (41). BR concentration was determined using a double beam spectrophotometer (U-3000, Hitachi) and a standard calibration curve, which was generated by adding known amounts of bilirubin to assay buffer followed by subsequent extraction. The detection limit of BR using this method was determined as 5 pmol BR. In all tissues the BVR activity (the capacity to convert BV into BR) was much higher (i.e., 10 times) than that of HO (the capacity to convert heme to BR). This indicates that BVR activity is not limited and that all BV formed by the HO enzyme is completely reduced to BR by the underlying BVR. Enzyme activities were expressed as pmol BR formed per mg protein in 30 min.

### Analysis of HO Protein Levels by Immunoblots

Proteins from droplets were separated under reducing conditions by sodium dodecyl sulfate polyacrylamide gel electrophoresis (SDS-PAGE) on 10–15% PAGE gradient gels and blotted onto nitrocellulose as described earlier (40). Immunoprobing with two specific antibodies was performed: against HO-1 (Alexis Corp., Lausen, Switzerland) and HO-2 (Santa Cruz Biotechnology, Santa Cruz, CA, USA), followed by cross-absorbed anti-rabbit IgG-HRPO (Novex, Life Technologies Corporation, Grand Island, NY, USA) and enhanced chemiluminescent detection (ECL-detection). Fluorescence staining of the overall protein pattern (prior to immunostaining) was used as a loading control and for normalization (Overall protein stains are shown in Figure S1). HO-1 proved to be below detection limit in brain. Testing of Hc and mC specimens for HO-2 was performed on individual animal basis.

### Analysis of Gene Expression

Gene expression analysis was performed using qPCR as described elsewhere (42). Briefly, RNA was isolated from 2 frozen droplets using 1 mL of TriReagent™. Extraction of RNA was performed in accordance to manufacturer's protocol. The amount of extracted RNA was determined spectrophotometrically at 260 nm and purity was assessed by the 260/280 nm ratio on an Eppendorf BioPhotometer plusUV/VIS (Eppendorf, Hamburg, Germany). Copy DNA was prepared as previously described (43). Equal aliquots from each cDNA were pooled to generate an internal standard (IS) which was used as reference for the quantification. Primer pairs used for the expression analysis of HO-1 and TNFR1, and for the internal reference genes *hypoxanthine ribosyltransferase* (HPRT), and *cyclophilin A* (Cyc) were previously published (42). Primer pairs for the analysis of HO-2 and BVR *isoform A* (BVRA) gene expression were newly established for this study. Results of validation experiments performed to verify the suitability of these qPCR assays in accordance to the MIQE guidelines (44) are available as Tables S4, S5 and Figure S2. The qPCR was carried out on a CFX96™ (Bio-Rad, Hercules, CA, USA). Data were analyzed using the inbuilt software CFX manager (Version 2.0, Bio-Rad) in the linear regression mode. Expression of target genes was calculated against IS using a modified ΔΔCq method and normalized for the relative expression values obtained for internal reference genes HPRT & Cyc as previously described (45). Values obtained from duplicates were averaged and expressed as 2^−ΔΔCq^ in fold changes relative to the IS.

### Histological Analysis

Fixed brain hemispheres were cut into coronary sections, which were embedded in paraffin wax and cut into 5 μm thick sections. Sections containing Hc, mC, striatum and cerebellum were stained with hematoxylin and eosin (HE) and examined by a pathologist blinded to the study groups. The presence of neuronal necrosis was determined in a descriptive manner in HE stained sections of these brain regions.

Immunohistochemistry was used to determine activation of microglia (primary antibody against ionized calcium-binding adapter molecule 1 (Iba-1), FUJIFILM Wako Chemicals, Neuss, Germany, dilution 1:80,000) and astrocytes (primary antibody against glial fibrillary acidic protein (GFAP), Agilent Dako, Waldbronn, Germany, dilution 1:5,000) in the Hc in a semiquantitative manner. Furthermore, immunohistochemical investigations using a primary antibody against HO-2 (Santa Cruz Biotechnology, Santa Cruz, CA, USA, dilution 1:100) were performed to evaluate expression of HO-2 in Hc and mC. All immunohistochemical stainings were done automatically on an autostainer (Lab Vision AS 360, Thermo Fisher Scientific, Waltham, MA, USA). Briefly, sections were cut and antigen retrieval was performed in the Lab Vision PT Module (Thermo Fisher Scientific, Waltham, MA, USA) with citrate puffer (pH6, Iba1, and HO-2) and pronase digestion (GFAP), respectively. Endogenous peroxidase activity was blocked by incubation in H_2_O_2_. Ultra Vision Protein Blocking reagent (Labvision/Thermo Fisher Scientific, Fremont, CA, USA) was used to avoid non-specific binding of antibody. After application of the primary antibody a polymer detection system (Ultra Vision LP Large Detection System HRP, Labvision/Thermo Fisher Scientific, Fremont, CA, USA) consisting of a secondary antibody formulation conjugated to an enzyme-labeled polymer was used. The polymer complex was visualized with diaminobenzidine (Labvision/Thermo Fisher Scientific, Fremont, CA, USA). Subsequently, sections were counterstained with hematoxylin, dehydrated and mounted with Neo-Mount (Merck, Darmstadt, Germany).

For the semiquantitative analysis of gliosis and expression of HO-2, the region of interest was evaluated in the respective immunohistochemical staining. An increase or decrease of staining intensity and extent was assessed on a five-point scale. Normal expression of Iba1 (microglia) and GFAP (astrocytes) was assessed as “0,” while an increase was assigned to the following categories: scattered (1), mild (2), moderate (3), or high (4) expression. For HO-2 expression, the normal expression pattern was rated as “4.” The staining intensity was compared to the adjacent cortex tissue and a decrease was rated in four categories: mild (3), moderate (2), severe (1) reduction or no signal at all (0). Adobe Photoshop CC 2019 was used for white balance and to assemble representative histological pictures.

### Statistics and Data Analysis

Data from quantitative analysis were calculated as medians as is recommended for qPCR data (46). Correlation between data sets was analyzed using the Spearman ranked sign test. Groups were compared by one-way non-parametric ANOVA (Kruskal Wallis) followed by Bonferroni-correction using IBM SPSS statistics (version 24). Values with *p* < 0.05 were considered significantly different. For visualization of data GraphPad PRIMS version 5.00 (GraphPad Software Inc.) was used, indicating the values of the single animals, and medians with 1st and 3rd quartiles per group. The numbers of independent samples (*n*) are indicated in the respective figure legends.

## Results

Of 32 enrolled rats, 9 rats had to be excluded from the study (Table 1). One of the 10 sham operated animals woke up during the surgery, anesthesia was deepened immediately and the rat survived (day 1 and 14: OPC 1; NDS 0%). This animal was excluded from final examinations. Three rats of the 6 min CA group and 5 of the 8 min CA group either had no ROSC or survived only few hours (6 min CA group: no ROSC, *n* = 1; OPC 5, *n* = 2; 8 min CA group: no ROSC, *n* = 2; OPC 5, *n* = 3). These animals were excluded from final examinations as well. A total of 23 animals (sham group, *n* = 9; 6 min CA group, *n* = 7; 8 min CA group, *n* = 7) remained for final examinations on day 14. From one rat of the 8 min CA group brain sample volume harvested was not sufficient for the biochemical analysis, only histological examinations and neurological scoring were performed. Mean arterial pressure and blood gas analysis at baseline (after surgery) and 5 min after ROSC are presented in Table S5. All surviving animals were scored for their neurological performance (OPC and NDS) at day 1 and at day 14 prior to sacrifice. We used repeated scoring as a suitable approach to rate the neurologic recovery, as was described earlier (7, 9). The results of the neurological scoring of CA animals with ROSC, including those with OPC 5 (= dead) at day 1, and the results obtained for animals surviving until day 14 are presented below (Table 2).

**Table 1:** Overview of animals enrolled in the study (*n* = 32).

**State of the animal**	**Sham**	**6 min cardiac arrest**	**8 min cardiac arrest**
Enrolled	10	10	12
Excluded from final examination	1	3	5
No return of spontaneous circulation (ROSC)	–	1	2
ROSC with overall performance category score = 5	–	2^a^	3^a^
ROSC with overall performance category score <5	–	7	7
14-day survival	9	7	7^b^

a *Rats died before the end of the experiment and were excluded from final examination*.

b *From one animal only histological examinations and no biochemical analysis were performed (see above)*.

**Table 2:** Results of repetitive neurological scoring of experimental animals.

**Neurological state**	**Day 1**			**Day 14**		
	**Sham**	**6 min CA^a^**	**8 min CA**	**Sham**	**6 min CA**	**8 min CA**
OPC^b^ 1	9	5	–	9	7	7
OPC 2	–	2	7	–	–	–
OPC 3	–	–	–	–	–	–
OPC 4	–	–	–	–	–	–
OPC 5 (dead)	–	2	3	–	–	–
NDS^c^ (OPC 1–4)	0 ± 0	5 ± 2	13 ± 4	0 ± 0	0 ± 0	1 ± 2

a *CA, cardiac arrest*.

b *OPC, overall performance category score (OPC 1 = normal; 2 = moderate disability. 3 = severe disability; 4 = comatose; 5 = dead; for details of calculating OPC see Table S2)*.

c *NDS, neurological deficit score (NDS 0% = normal; NDS 100% = dead; mean ± SD; for details of calculating NDS see Table S3)*.

### CA Leads to Decreased Activities of HO in Motor Cortex and Hippocampus, While BVR Activities Are Not Affected

**Figure 3:**
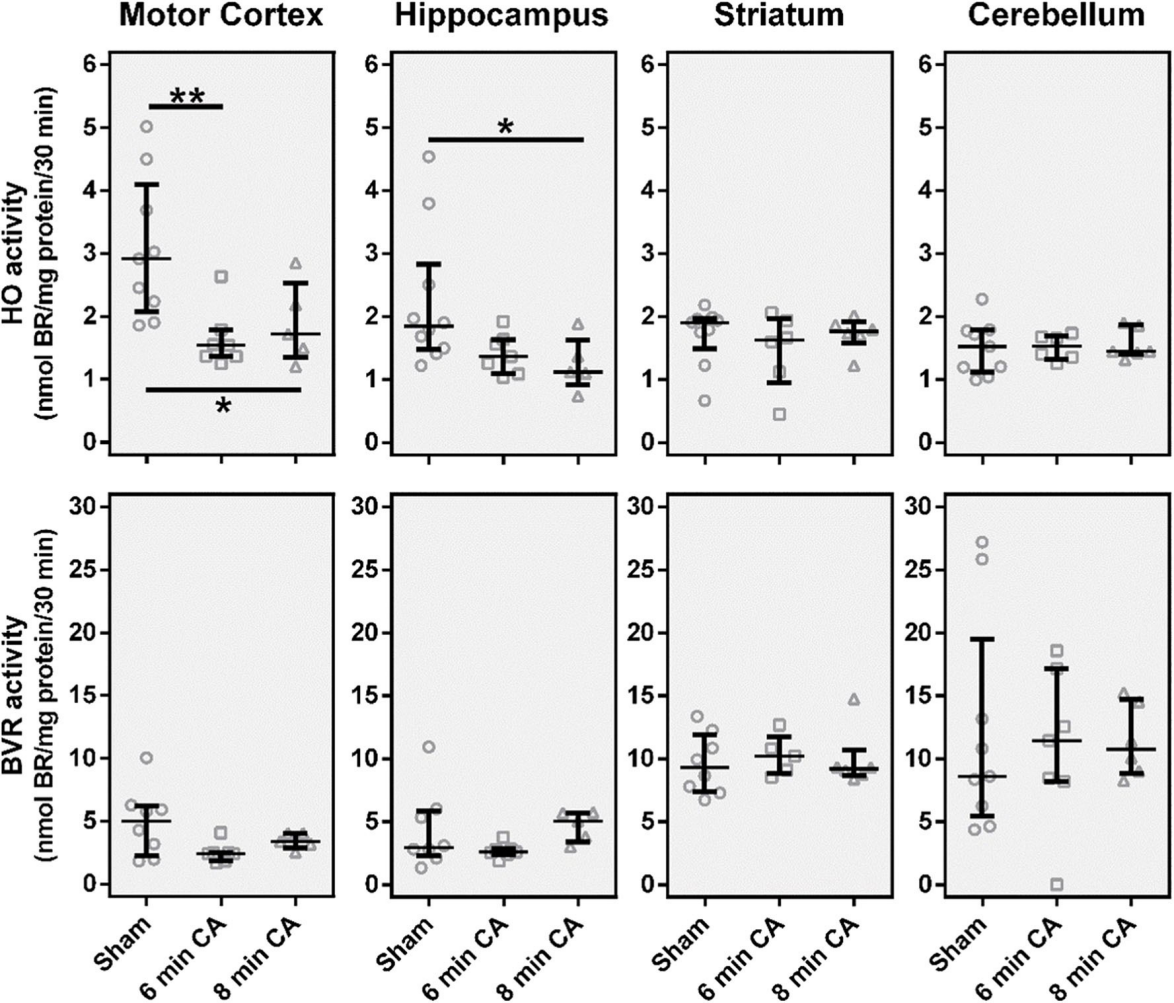
Catalytic activities of the heme degradation pathway enzymes, HO and BVR, in brain regions 2 weeks after cardiac arrest (CA) (6 or 8 min) and resuscitation. Animals were subjected to CA for 6 min (CA 6 min) or 8 min (CA 8 min) or sham operated, as outlined in Materials and Method section. Brain regions were analyzed for enzyme activity by measuring the capacity of homogenized tissue to convert heme (HO activity) or biliverdin (BVR activity) into bilirubin within 30 min. The obtained amount of BR was corrected for the underlying protein concentration and enzyme activity is given in nmol BR formed per mg protein in 30 min (nmol BR/mg protein/30 min). Data are shown for single animals (gray open symbols) in each group: sham (instrumented animals, open circles, *n* = 9), rats subjected to CA for 6 min (CA 6 min, open squares, *n* = 7) or 8 min (CA 8 min, open triangles, *n* = 6), indicating additionally group medians (thin black line) and 1st and 3rd quartiles (bold black lines). Differences between groups are indicated (one-way non-parametric ANOVA (Kruskal Wallis) Bonferroni-corrected; *p < 0.05; **p < 0.01).

From all investigated tissues we found HO activity to be lower in samples from mC and Hc, while striatum and cerebellum did not show changes (Figure 3), when compared to the sham control group. For the mC, the median of the 6 min CA group was decreased by 47% (*p* = 0.009) and of the 8 min CA group by 42% (*p* = 0.043). In the Hc, the activities were decreased to a lesser extent [6 min CA, 26% (*p* = 0.136); 8 min CA, 39% (*p* = 0.029)]. BVR activity was not affected in any brain region (Figure 3).

### Decreased HO-2 Protein Levels in Response to CA Occur in mC (Immunoblot) and Hc (Immunohistochemistry)

**Figure 4:**
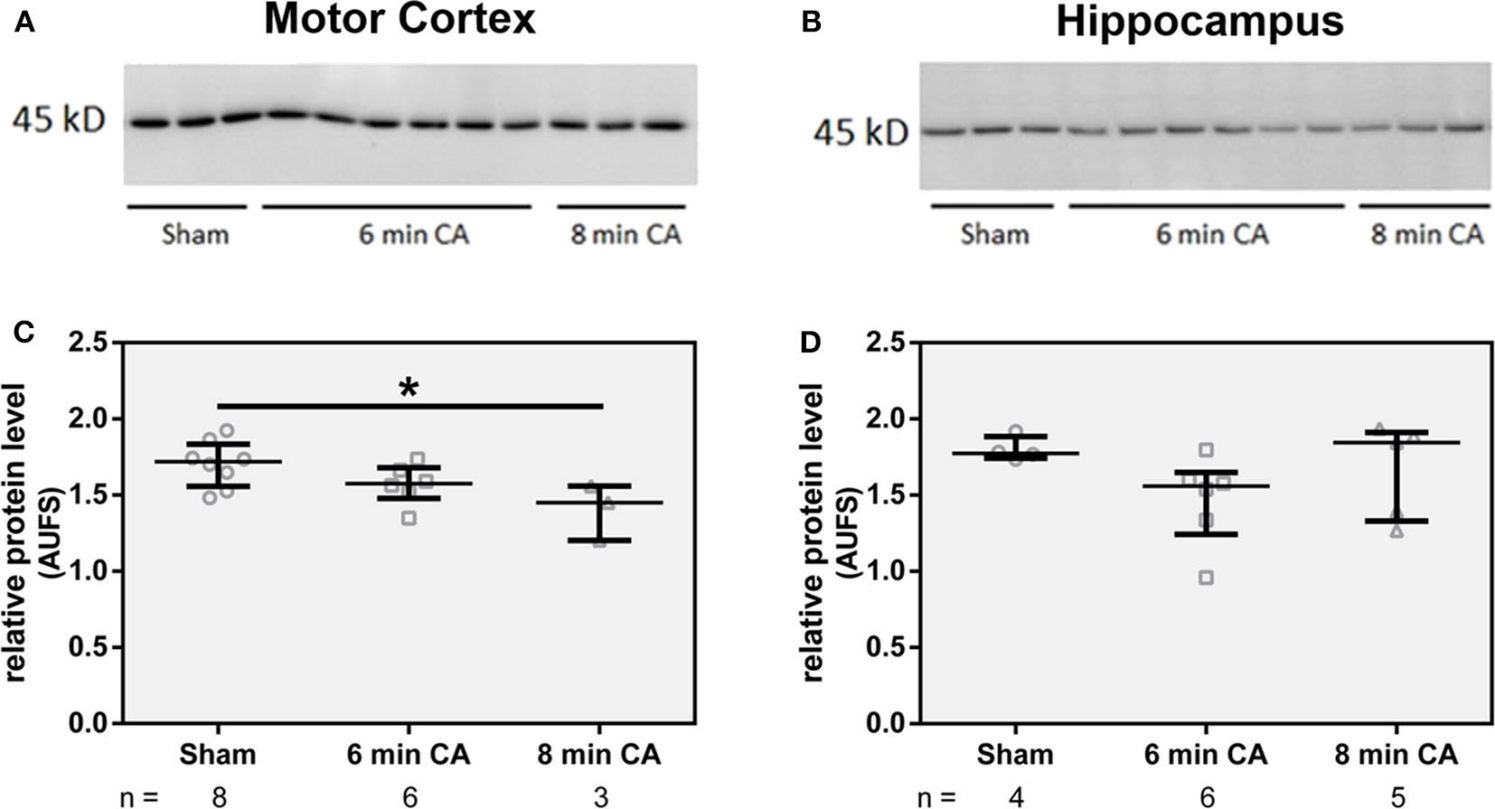
Abundance of HO-2 in homogenates of mC **(A)** and Hc **(B)**. Homogenates from brain regions of single animals were analyzed by SDS-PAGE and immunostained for HO-2 as described in Materials and Methods section. For quantification, band intensities of HO-2 specific staining were normalized to total protein staining of the respective gel lanes, in **(C)** for mC and in **(D)** for Hc. Values are given as AUFS (arbitrary units) as single data (gray open symbols) for each group: sham animals (open circles), rats subjected to cardiac arrest (CA) for 6 min (CA 6 min, open squares) or 8 min (CA 8 min, open triangles), indicating additionally group medians (thin black line) and 1st and 3rd quartiles (bold black lines). The numbers of analyzed animals per group are indicated below the graphs; a selection is displayed here. Total protein patterns of the respective gels are shown in Figure S1. Differences between groups are indicated (one-way non-parametric ANOVA (Kruskal Wallis) Bonferroni-corrected; *p < 0.01).

Since we have found lower HO activity in mC and Hc homogenates, we questioned whether decreased levels of HO-2 protein caused this effect. Although both isoforms HO-1 and HO-2 contribute to the overall HO activity, the amount of HO-1 protein is generally very low in neuronal tissues (20). In line with these findings, we also could not determine HO-1 at protein level in immunoblots of tissue homogenates. Though, in homogenates of mC from CA animals we determined lower levels of HO-2 protein (*p* = 0.01 for 8 min CA; Figures 4A,C). Additionally, homogenates of Hc from 6 min CA animals displayed slightly lower HO-2 protein levels in comparison to the respective sham animals, but this difference was not significant (*p* = 0.233; Figures 4B,D). It is possible that the differences between groups in Hc did not result in statistical significances, because of the low sample numbers available for some of the groups. We also tested the immunohistochemical appearance of HO-2 specific staining in sections of both brain regions (mC and Hc).

**Figure 5:**
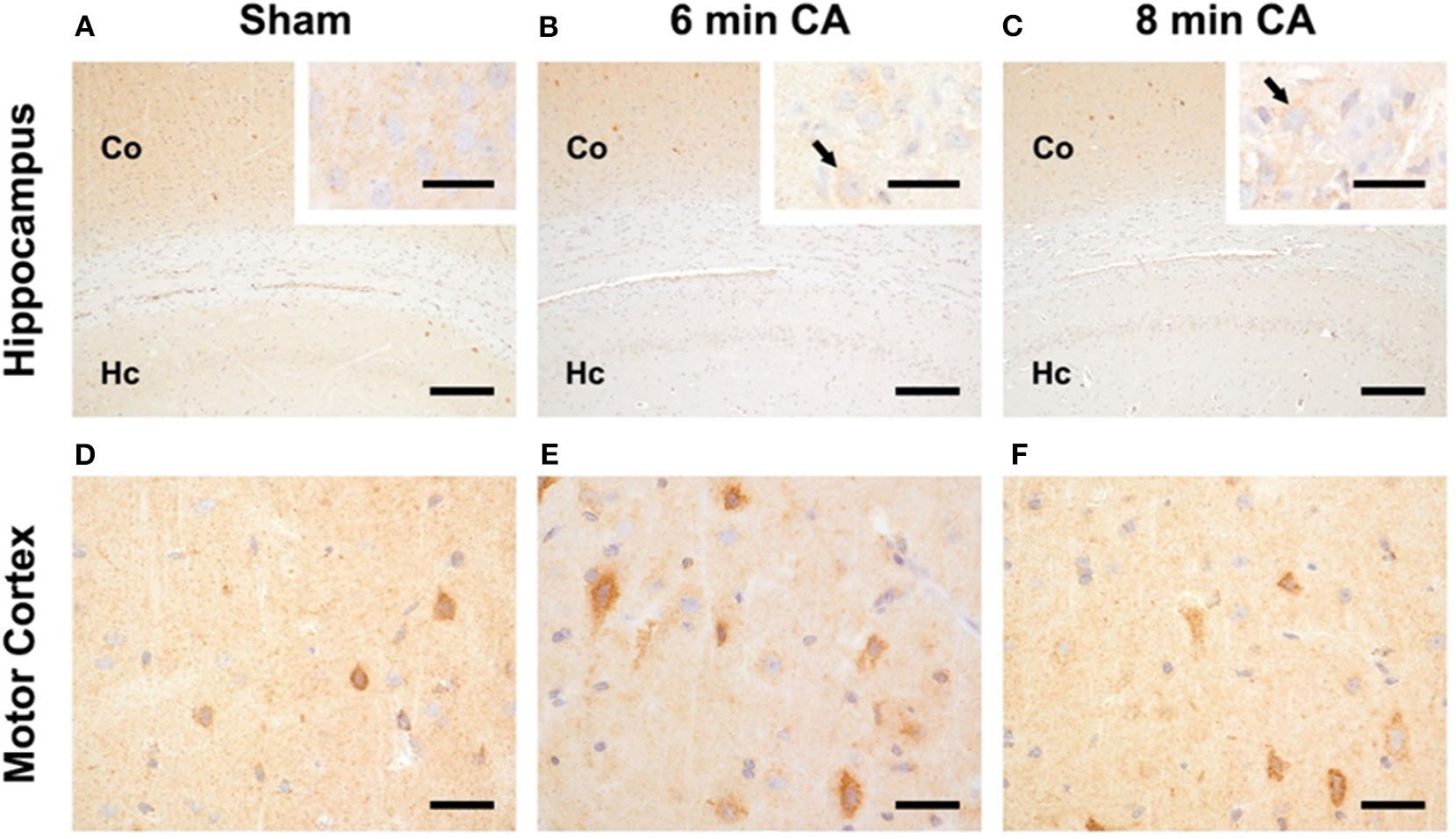
Representative pictures of HO-2 expression in hippocampus and motor cortex. **(A–C)** Expression of HO-2 in the Hc, bar = 30 μm, bar in inserts = 150 μm. **(A)** The signal intensity in the hippocampus (Hc) of sham animals is nearly similar to the expression in the respective overlying cerebral cortex (Co). **(B,C)** Reduced expression of HO-2 in the Hc of 6 and 8 min CA animals compared to the respective cerebral cortex and compared to sham animals. Inserts: HO-2 expression is detectable in all pyramidal neurons of sham animals **(A)**, but only in viable pyramidal neurons [arrows in **(B,C)**] in CA animals. **(D–F)** Expression of HO-2 in the mC, bar = 30 μm; consistent expression of HO-2 in sham **(D)** as well as 6 **(E)** and 8 min **(F)** CA animals with increased staining intensity in some neurons.

In sham animals, immunohistochemical staining for HO-2 showed a consistent signal in all layers of the Hc *CA1*-region (Figure 5A). The signal intensity was nearly similar to the signal intensity in the cerebral cortex in the same section (Figure 5A). In contrast, in all CA animals, regardless of CA duration, the HO-2 signal was significantly reduced in all hippocampal layers compared to the corresponding cerebral cortex (Figures 5B,C and Table 3). The neuronal expression of HO-2 was present predominantly in surviving pyramidal neurons in CA animals (Figures 5B,C inserts). In all animals, a consistent signal was present in the neuropil of the mC, and in some neurons increased staining intensities were detectable (Figures 5D–F). In contrast to Hc, in mC, the HO-2 specific staining was heterogeneous and cell type-specific, making quantitative considerations with this technique difficult. Nevertheless, the overall protein, determined by immunoblots, revealed that CA decreased HO-2 levels in mC as well. Therefore, our data suggest that the decreased HO activity may be caused by lower levels of HO-2 protein in mC and Hc.

**Table 3:** Semiquantitative evaluation of HO-2 and markers indicative for activation of astrocytes and microglia in hippocampal sections of rats after 2 weeks following cardiac arrest.

**Marker**	**Sham**	**6 min CA^a^**	**8 min CA**
HO-2	4 (4,4)	2 (1,2)^***^	1 (12)^***^
Iba1^b^	0 (0, 0)	3 (3,3)^***^	3.5 (3,4)^***^
GFAP^c^	0 (0, 0)	3 (2,4)^***^	4 (3,4)^***^

a *CA, cardiac arrest*.

b *Iba1, ionized calcium-binding adapter molecule 1 (microglia activation marker), activation of*.

c Glial fibrillary acidic protein; data are given as mean ± SD. Details of the scoring system yielding median and interquartile ranges are described in the respective Materials and Methods section. Significant differences were detected between sham animals and animals subjected to CA for all markers (

*** *p < 0.005 vs. sham), but not between 6 and 8 min CA animals*.

### Gene Expression Level of Enzymes of the Heme Degradation Pathway and Activation of Inflammatory Pathways (TNFR1)

**Figure 6:**
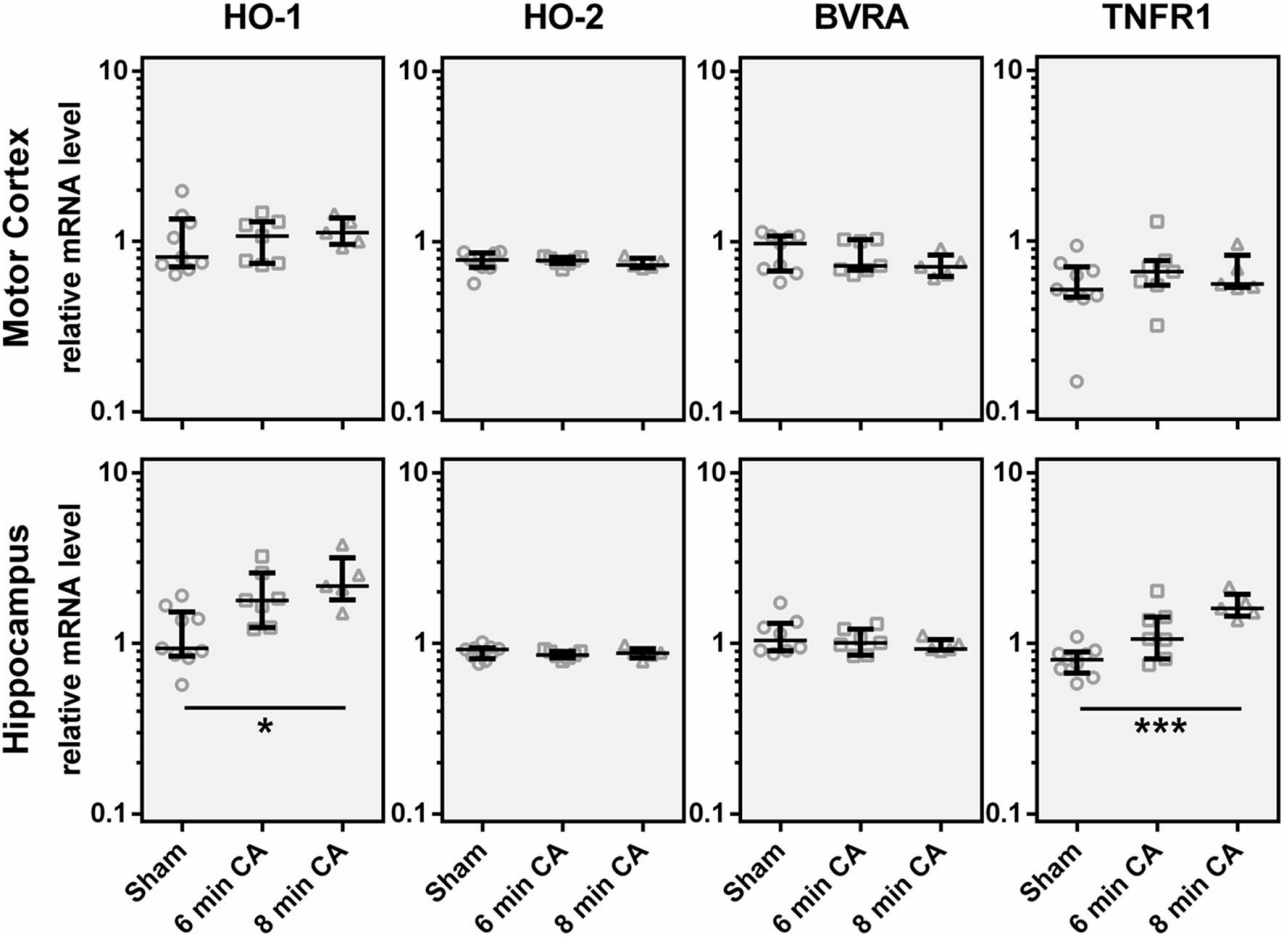
Expression of, HO-1, HO-2, BVRA and TNFR1 mRNA in motor cortex and hippocampus 2 weeks following cardiac arrest (CA). Gene expression was quantified by qPCR. Data were normalized against the internal reference genes HPRT and *Cyclophilin A* and expressed relative to the IS (relative mRNA level). Gene expression levels are shown for single animals (gray open symbols) in each group: sham animals (open circles, *n* = 9), rats subjected to CA for 6 min (CA 6 min, open squares, *n* = 7) or 8 min (CA 8 min, open triangles, *n* = 6), indicating additionally group medians (thin black line) and 1st and 3rd quartiles (bold black lines). Significant differences between groups were calculated by one-way non-parametric ANOVA (Kruskal Wallis followed by Bonferroni-correction) and are indicated (*p < 0.05; ***p < 0.005).

In order to find out whether the decrease in HO-2 protein was caused by a decreased mRNA transcription, we determined gene expression levels. Additionally to HO-2 mRNA, we also analyzed HO-1 and BVRA mRNA levels to cover gene transcription of all enzymes of the heme degradation pathway. Contrary to our expectation, we did not find gene expression levels of either HO isoform decreased. In contrast, the expression levels of HO-1 were increased in Hc of rats subjected to 8 min CA (Figure 6). The HO-1 expression levels showed an association with the duration of CA, since highest levels were found in the 8 min CA group. The median of the 6 min CA group showed an increase of 91%, while the median of the group of animals with 8 min CA showed an increase of 132%. Neither HO-2, nor BVRA mRNA showed any changes (Figure 6).

It is known that HO-1 (synonym HSP32) is indicative for the activation of a stress response. Activation of astroglia is frequently associated with an increased HO-1 expression, along with other inflammation associated markers, such as TNFR1. Indeed, we could also show upregulation of TNFR1 mRNA in Hc of rats subjected to 6 and 8 min of CA (Figure 6). The expression levels of TNFR1 mRNA showed a significant correlation with HO-1 mRNA (Spearman Rho, *R* = 0.649; *p* = 0.001, data not shown). The median of the 6 min CA group showed an increase of 33%, while the median of the 8 min CA group showed a more than two-fold increase. Compared to the sham animals this increase was significant for the animals subjected to 8 min of CA (*p* = 0.003), but only by trend for the animals subjected to 6 min of CA (*p* = 0.055). None of the other regions displayed increased levels of TNFR1.

### Histopathological Changes Only in Hc 2 Weeks After CA

The results of mRNA expression analyses suggested an ongoing stress response and additionally the number of HO-2 expressing neurons was diminished in Hc. In order to determine the morphological appearance of lesions in response to CA and reperfusion we evaluated HE-stained sections of all investigated regions.

**Figure 7:**
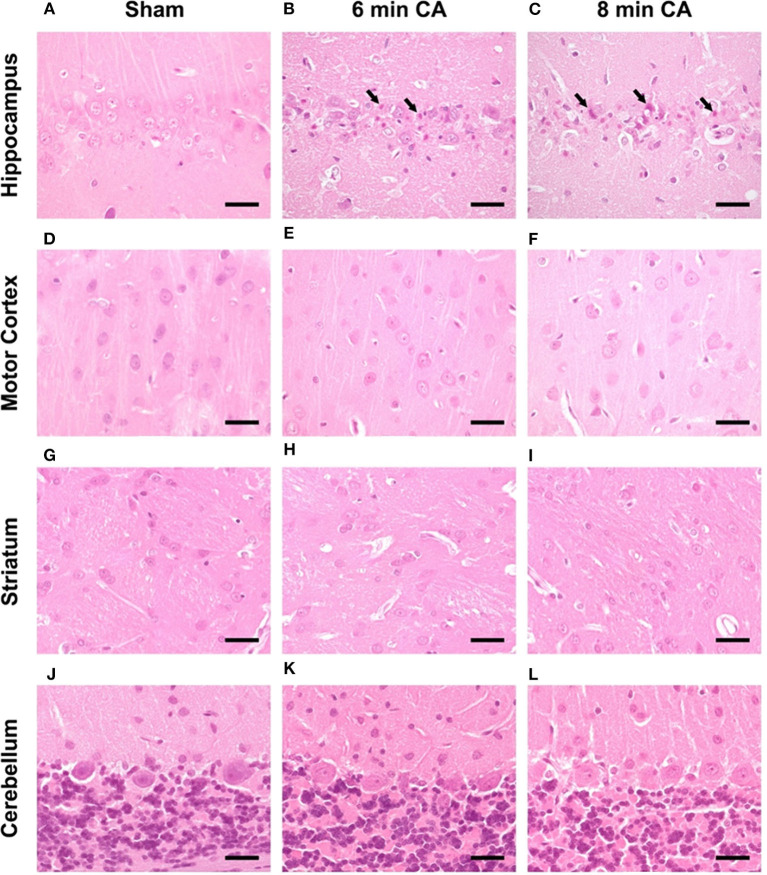
Representative pictures of hippocampus, motor cortex, striatum and cerebellum at 14 days after CA, HE-staining, bar = 30 μm. **(A–C)** Hippocampus, **(A)** sham animal with viable pyramidal neurons, no lesions present. **(B)** 6 min CA animal with many necrotic neurons (arrows) and few viable pyramidal neurons; **(C)** 8 min CA animal with many necrotic neurons (arrows) and only scattered viable pyramidal neurons. **(D–F)** Motor cortex, viable neurons in sham **(D)** as well as 6 **(E)** and 8 min CA **(F)** animals, no lesions present. **(G–I)** Striatum, viable neurons in sham **(G)** as well as 6 **(H)** and 8 min CA **(I)** animals, no lesions present. **(J–L)** Cerebellum, viable neurons in sham **(J)** as well as 6 **(K)** and 8 min CA **(L)** animals, no lesions present. CA, cardiac arrest.

Consistent lesions were detectable only in the Hc of animals with CA. Sham animals did not show any lesions in the *CA1* region (Figure 7A). Hypereosinophilic pyramidal neurons with shrunken nuclei (necrotic neurons) were present in animals with 6 and 8 min CA (Figures 7B,C). Animals with 8 min CA showed a tendency toward more severe lesions, but no significant differences were found compared to 6 min CA animals. Lesions were not detected in mC (Figures 7D–F). Also striatum (Figures 7G–I) and cerebellum (Figures 7J–L) showed no lesions, regardless of CA duration and consistent with earlier findings (7).

### CA Leads to Activation of Microglia and Astrocytes in Hc

**Figure 8:**
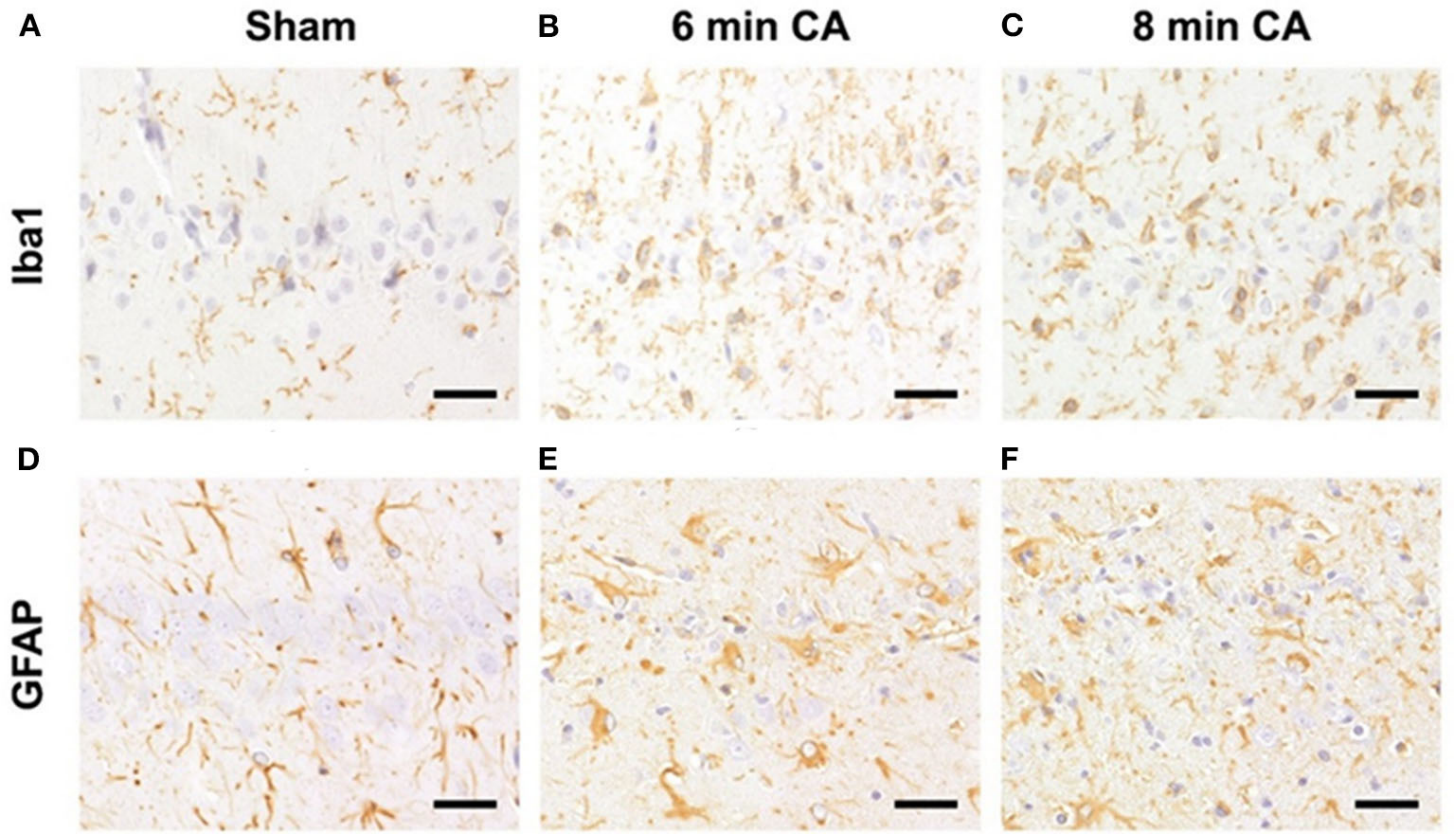
Representative pictures of microglia and astrocyte activation after CA in the hippocampus, bar = 30 μm. **(A–C)** Detection of microglia (antibody against Iba1) in the Hc. **(A)** Normal appearance and numbers of microglial cells in sham animal. **(B,C)** Increased numbers of activated microglia in CA animals. **(D–F)** Detection of astrocytes (antibody against GFAP) in the Hc. **(D)** Normal appearance and numbers of astrocytes in sham animal. **(E,F)** Increased numbers of gemistocytes in CA animals. CA, cardiac arrest.

Hc showed loss of pyramidal neurons in association with increased mRNA expression levels of inflammatory markers in animals subjected to CA surviving 14 days. We therefore aimed at confirming ongoing gliosis by additionally analyzing typical markers. Using immunohistochemistry we determined an increased staining of Iba1 and GFAP regarding extent and intensity in microglia (Figures 8A–C) and astrocytes (Figures 8D–F) in CA-animals. Microglia showed rod-shaped nuclei and soma with short thick processes consistent with activation. Cytoplasm and nuclei of astrocytes were swollen with large thick processes showing gemistocytic appearance. By semiquantitative evaluation of glial activation statistically significant differences were detected between sham and CA animals in Iba1 and GFAP immunohistochemistry (*p* < 0.005). However, no differences were present between 6 and 8 min CA groups (Table 3).

## Discussion

We could show that 2 weeks after CA hippocampal and motor cortex tissues display decreased HO activity, which is a reduced capacity to convert heme. Although several reports have brought evidence for the relevance of HO in protecting susceptible neuronal structures against the consequences of ischemia, a complete assessment of region specific changes of the cerebral HO system in response to CA is not available yet.

Ischemia as a result from CA and resuscitation result in the well-described phenomenon of ischemia/reperfusion injury (47). Neuronal cells display a particular vulnerability, which depends primarily on the duration of the ischemic insult, but also on the brain region itself (48, 49) and *CA1* pyramidal neurons of the Hc appear consistently affected.

We found that 2 weeks after CA pyramidal neurons in the *CA1* region were lost and staining intensity of Iba1 and GFAP were increased, showing activation of microglia and astrocytes. In Hc, we found higher mRNA levels of HO-1, which correlated with TNFR1 mRNA, both markers of inflammatory cell stress. The degree of neurodegeneration seen in Hc showed a tendency to increase with the duration of CA, since animals subjected to 8 min of CA displayed changes that were more pronounced. Our findings confirm that cerebral ischemia affects nearly always the Hc region (15), in which neuroinflammation and neurodegeneration is persisting over long periods (17, 50).

Loss of pyramidal neurons in *CA1* is known to lead to learning and memory deficits, and the reappearance of neurons in *CA1* to improved learning and memory performance (51). Meanwhile it is well-accepted that HO plays an important role for survival of neurons, including *CA1* neurons of Hc in response to cell stress, such as ischemia. A model of asphyxia induced cardiac arrest showed that neuronal injury and neuronal loss in the *CA1* region were lower after 2 weeks when animals were pre-treated with heme, while neuronal loss was higher upon pre-treatment with a HO inhibitor (52). However, neither expression levels, nor activities of the enzymes of the heme degradation pathway were analyzed in the cited study. Nevertheless, a recent study suggested a close association of cognitive capabilities and the actual HO activity in Hc. Reversal of age-related cognitive deficits and neuronal loss in *CA1* was associated with an increase in heme degrading capacity of Hc and frontal cortex (53).

Functional HO enzyme maintains cellular homeostasis and protection of neurons by different means (54). HO reaction products play an important role in stress defense and tissue regeneration (30–32) and transiently increased HO activity therefore supports neuronal repair and regeneration following an insult (55). Decreased HO activity, in contrast, impairs neuronal function and aggravates neuronal injury. Deletion of HO-2 in neuronal cells of Hc and cortex resulted in oxidative stress mediated injury, which was absent in cells with functional HO-2. The neuroprotection was attributed to BR (56), which may protect cells against a 10,000-fold excess of H_2_O_2_ (57). Further, neuronal function requires CO (58), particularly that of neurons in Hc (59). Thus, inhibition of HO within the Hc by pharmacological means resulted in retrograde amnesia (60), showing that active HO is required for memory consolidation. Memory consolidation involves cGMP (21), a second messenger formed by soluble guanylate cyclase, of which CO, a product of the HO reaction, is a known activator.

The decrease in HO activity, which we have determined in mC and Hc tissue, is supposed to operate simultaneously in two adverse directions: on one hand, it will result in (i) heme accumulation and on the other hand in (ii) a decreased generation of HO reaction products (see below). Excess free heme is highly toxic, especially for neuronal cells, due to its ability to promote oxidative stress (61). Based on its lipophilic nature, heme may induce lipid peroxidation and subsequent membrane injury, which finally results in apoptosis. Indeed, scavenging of heme by intracerebroventricularly applied hemopexin reduced the infarct volumes, improved neurological function and cognitive function after focal ischemia (62, 63). We therefore think that the decreased HO activity, which is caused by CA, results in neuronal deficits.

Up to now, it is not possible to determine the contribution of the single HO isoforms to the overall heme degrading capacity in tissue homogenates, because isoform specific inhibitors are not available. However, it is known that in neuronal tissue nearly all of the HO-activity is ascribable to the constitutive HO isoform HO-2 (64), and the contribution of HO-1 is almost absent in physiological conditions (20). Accordingly, studies using HO-2 knockout mice showed that although traumatic brain injury dramatically upregulated HO-1, and HO activity was slightly increased shortly thereafter, the HO activity determined in injured mice remained far below the values found in wild type control mice (65). Only few studies, focusing on the events occurring shortly after the insult, investigated HO activity in neuronal tissues following ischemia. Spinal cord injury resulted in an increased HO activity few days later (66). In another model of focal cerebral ischemia injured areas displayed an increased HO-1 protein abundance 3 days later, which correlated with a locally increased capacity to produce BR (67). Thus, even though stressful conditions increase the concentration of HO-1 the contribution of this isoform to the overall HO activity appears limited and may be timely restricted in neuronal tissues. This interpretation is supported by the fact, that HO-1 protein was not detectable in mC and even in Hc, despite the increased mRNA expression. Therefore, we can assume that HO-1 protein did not contribute noteworthy to the measured HO activity.

In contrast, our findings suggest that the decreased HO activity in rats subjected to CA, results at least partially from lower HO-2 protein concentrations in mC and Hc tissue. The *in-situ* protein expression of HO-2 in both regions using immunohistochemistry showed that HO-2 expression varies among cell types. Neuronal cells displayed stronger staining intensities, and highest HO-2 levels were found in selected neurons in mC. Due to this heterogeneity, HO-2 quantification by immunohistochemistry is difficult. However, the overall quantification using western blots showed a decrease in HO-2 protein in mC, which was significant, when CA lasted 8 min. Although we could not find morphological signs for neuronal loss in mC a loss of a few of these HO-2 highly positive cells is supposed to contribute measurably to a decrease in the overall protein and enzyme activity. Compared to mC, in Hc HO-2 staining was more homogenous, enabling a semiquantitative approach using immunohistochemistry. In rats subjected to CA, HO-2 representation was lower in all layers of Hc. This difference became obvious, when comparing the staining intensity with the adjacent cortex tissue. Further, HO-2 staining was predominantly present in viable pyramidal neurons of *CA1*. Since these neurons are a significant source of HO protein, it is highly probable that a loss of these cells caused the decrease in HO activity. Unfortunately, our data obtained from HO-2 immunoblots of Hc tissue homogenates did not reveal significant differences among groups, possibly because not all animals were included into this analysis, due to the limited amount of tissue material. Nevertheless, using different approaches, our data suggest that the decreased HO activity in mC and Hc of rats subjected to CA may result from lower levels of HO-2 protein present in these regions.

We assume that this decrease in HO-2 was not caused by gene regulation, since HO-2 mRNA expression was unchanged in both regions, and in Hc, HO-1 mRNA was even increased in response to CA. We can rule out that the partially opposing results, which we have obtained, are caused by area specific effects associated with heterogeneous cellular composition and architecture of neuronal tissue. Our methodological approach of using homogenates of the entire regions of interest, allowed to directly compare the obtained data for all quantitative parameter. Probably, CA induced a loss of HO-2 positive cells.

Additionally, it is possible that posttranslational modifications that may affect the enzymatic activity (33, 68) are involved. Oxygen and nitrogen radicals induce HO-1 mRNA, but simultaneously, they may induce posttranslational protein modifications, which down-modulate HO-activity (68). Hippocampal tissue of aged subjects with cognitive impairment or Alzheimer disease display increased levels of HO-1 protein showing oxidative posttranslational modifications (33, 69). Further, amyloid precursor proteins may associate with HO (70), inhibit HO activity and thereby increase oxidative stress levels, attributed to a decreased production of BR (34). Interestingly, amyloid-β peptide is an integral part of the cGMP-induced memory induction (71) that requires functional HO enzyme. These findings suggest the existence of a vicious cycle consisting of oxidative stress induced HO-1 expression and a lower HO activity due to chronically enhanced oxidative stress levels.

Further studies are needed to clarify the cause for the decrease in HO activity; however, our findings suggest compromised neuronal function in both regions, Hc and mC. Additionally, the decreased HO activity in Hc may compromise the repair mechanisms and prevent reduction of oxidative stress levels. We think that the increased HO-1 mRNA levels in Hc seen at this late time point after CA stands for the attempt to compensate for the decreased HO activity in order to restore tissue homeostasis. Not necessarily, an increased HO activity must result thereof. In contrast, many studies show that changes in mRNA or protein levels also for other enzymes relevant for the antioxidant defense do not fully account for the changes in enzyme activity determined under certain stress conditions (72, 73). Thus, contrary to the general assumption, HO mRNA (and protein) expression levels are not suitable to predict the resulting HO activity in neuronal tissues.

Except for Hc, our model revealed absence of histological signs for neurodegeneration or gliosis at this late time point (7, 9, 74). It has been shown, that the duration and the depth of the ischemic insult affect the chronology of the manifestation of neuronal damage in the different brain regions (75). Although we found decreased HO activity in mC, this region did not show morphological signs for neurodegeneration. However, mC might functionally respond to CA and the decreased HO activity may indicate a slowly progressing neuronal dysfunction, which is not leading to such an impressive loss of neurons, as is the case for Hc. However, further studies are required to elucidate the mechanism underlying the decrease in HO activity, which may contribute to the delayed neurodegeneration in Hc and mC after CA.

## Conclusion

Our data revealed decreased HO activity in two brain regions, namely Hc and mC in a clinically relevant model for human ventricular fibrillation cardiac arrest and resuscitation 2 weeks after global ischemia. Our data suggest that reduced protein levels of HO-2 may contribute to the decreased tissue capacity to produce HO reaction products. In the *CA1* region of Hc, a region typically affected in cerebral ischemia, the decrease in HO activity went in parallel with neuronal loss in *CA1* and an increase in levels of markers indicating ongoing gliosis. Although the decreased HO activity in mC was not associated with visible lesions, HO-2 positive cells may have been lost in response to CA. Considering the importance of HO for neuronal protection and function, it is conceivable that decreased HO activity is causally involved in the delayed neurodegenerative processes, which contribute to neuronal dysfunction frequently occurring in CA patients. Our findings further suggest that protein or RNA expression data do not allow inferring HO activities in neuronal tissues.

## Data Availability Statement

All datasets presented in this study are included in the article/Supplementary Material.

## Ethics Statement

The animal study was reviewed and approved by Committee of the Medical University of Vienna and the Austrian Ministry of Science, Research and Economy (GZ: 66.009/0064-II/3b/2011).

## Author Contributions

A-MW performed animal experiments and wrote the first draft of the manuscript. JH prepared and analyzed samples and performed the gene expression analysis with AM, FE, UT, IAM, BB, and A-MK and contributed with acquisition of data. AJ implemented the setup and procedures of the animal experiments and collected samples. IM and RM performed biochemical analysis and visualization of results. SH undertook the histological analysis and interpretation of results. JD performed the statistical analysis and prepared the final figures and tables. MH, FS, WW, and JD planned and designed the study. WW and JD wrote the final version of the manuscript. All authors approved the final version of the manuscript.

## Conflict of Interest

The authors declare that the research was conducted in the absence of any commercial or financial relationships that could be construed as a potential conflict of interest.
