# Supplementary material for: Motor Cortex and Hippocampus Display Decreased Heme Oxygenase Activity 2 Weeks After Ventricular Fibrillation Cardiac Arrest in Rats
Source: Front Med (Lausanne). 2020 Sep 10;7:513. doi: 10.3389/fmed.2020.00513 (PMC7511667; doi:10.3389/fmed.2020.00513)
Supplement: Supplementary file 1 [file Data_Sheet_1.pdf]

Supplementary Materials:

1) Animal performance

Table S1: Mean arterial pressure (MAP) and arterial blood gas analysis in cardiac arrest (CA) and sham animals after surgery (baseline) and return of spontaneous circulation (ROSC).

**Variables**	**Time Point**					
	**Baseline**			**5 minutes after ROSC**[1]		
	Sham (n=9)	6 min CA[2] (n=7)	8 min CA (n=7)	Sham (n=9)	6 min CA (n=7)	8 min CA (n=7)
MAP[3]	99±22	110±10	111±13	100±15	108±13	100±20
pH	7.42±0.02	7.41±0.04	7.40±0.03	7.42±0.02	7.09±0.05	7.05±0.08
$paCO_2$[4] (mmHg)	41±4	40±6	41±3	40±3	51±8	53±10
paO_2[5] (mmHg)	166±50	131±31	114±21	130±20	265±58	289±81
Hb[6] (g/dL)	14±1	14±1	14±1	14±0	15±1	15±1
K[7] (mmol/L)	4.1±0.5	4.2±0.6	4.3±0.4	4.2±0.4	4.4±0.2	5.5±0.8
Glucose (mg/dL)	170±26	183±42	166±21	168±21	334±26	347±38
Lactate (mmol/L)	1.3±0.5	1.4±0.6	1.1±0.1	1.4±0.2	10.5±1.2	12.2±0.8

[1] ROSC, return of spontaneous circulation; [2]CA, cardiac arrest; [3]MAP, mean arterial pressure; [4]$paCO_2$, arterial carbon dioxide partial pressure; [5]paO_2, arterial oxygen partial pressure; [6]Hb, hemoglobin; [7]K, potassium; (6 min CA, n=7; 8 min CA, n=7; Sham, n=9). Data are presented as mean ± standard deviation.

Table S2: Overall Performance Category (OPC) Score in rats. OPC 1 = normal; OPC 2 = moderate disability; OPC 3 = severe disability; OPC 4 = comatose; OPC 5 = dead. Abbreviations: + must be present; - is absent; ± may or may not be present.

OPC Score	Condition	Walk	Eat/drink	Motor	Sensory	Breath
1	normal	+	+	+	+	+
2	moderate disability	-	±	+	+	+
3	severe disability	-	-	-	+	+
4	comatose	-	-	-	-	+
5	dead	-	-	-	-	-

Table S3: Neurological deficit score (NDS) in rats. A normal rat has an NDS of 0 points (0%); a (brain) dead rat has an NDS of 100 points

Parameter		Score		Characteristics		
		Maximal points (worst condition)	Percentage (max 100%)	Description		
General deficit	Consciousness	20	20	20 (comatose)	10 (somnolent)	0 (normal)
	Respiration	20	20	20 (abnormal breathing)		0 (normal breathing)
Cranial nerve reflexes deficit	Olfactory (sniffing food)	4	4	4 (no reaction)		0 (normal)
	Vision (follows hand)	4	4	4 (no reaction)		0 (normal)
	Corneal reflex	4	4	4 (absent)		0 (present)
	Whisker Movement	4	4	4 (no whisker movement)		0 (normal)
	Hearing (turns to clapped hands)	4	4	4 (no reaction)		0 (normal)
Motor deficit	Motor	10	10	2.5 points for motoric problems for each affected extremity		0 (normal)
Sensory deficit	Sensory	10	10	2.5 points for sensory loss for each affected extremity		0 (normal)
Coordination deficit	Travel ledge	5	5	5 (no ability to walk on a beam)	2.5 (ability to walk on a beam with some help or for shorter periods)	0 (physiological behaviour)
	Placing test (front paws reaching when lifted from ground by tail)	5	5	5 (absent)		0 (present)
	Righting reflex (attempting to right self when placed on back)	5	5	5 (absent)		0 (present)
	Stop at table edge	5	5	5 (absent)	2.5 (animal recognises table edge, but is too weak to prevent falling down)	0 (present)

2) Protein loading controls.

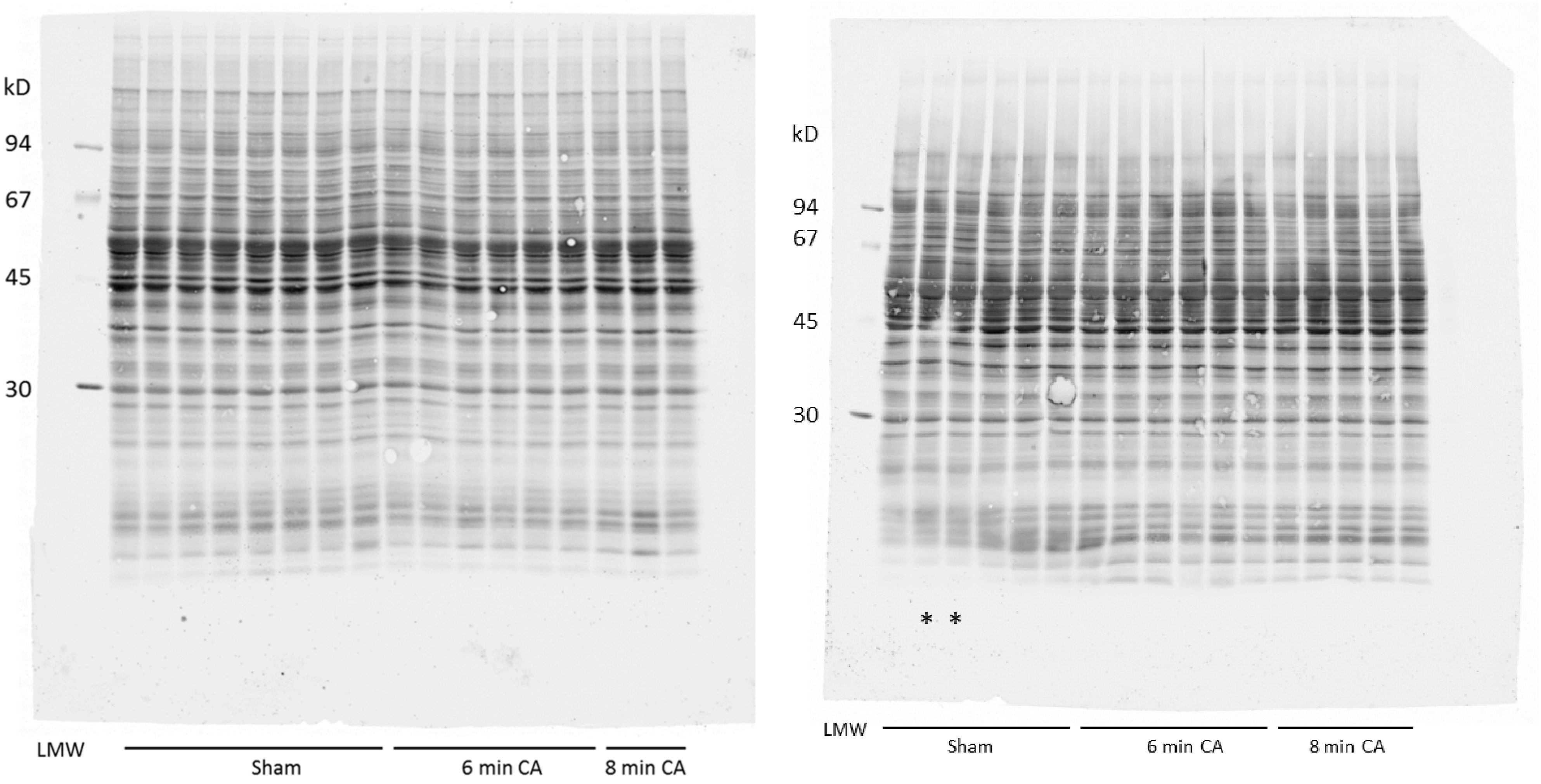

(A) (B)

Figure S1: Overall protein stain of samples separated by SDS PAGE. Group allocation is indicated by the bars beneath the lanes. (A) Samples investigated from mC homogenates. (B) Samples investigated from Hc homogenates. * These lanes were not used for quantitative evaluation.

3) Optimisation and validation of qPCR assay for HO-2 and BVRA in rat.

Table S4: Information about Intron-spanning newly established primers for HO-2 and BVRA.

Target	Accession number	Sequence	Position on plus-strand	Product-length (bp)	Exon junctions in	Intron size (bp)	Source
**HO-2**	NM_024387.2	GGG GAA GGG ACC CAG TTC TA	725	292	Product	677	newly designed
		CCA GGG TAC CTT TGT CTG GC	1016		Reverse primer	---	Primer BLAST
**BVRA**	NM_053850.1	CAT GTC CTC GTG GAA TAC CC	374	186	Product	5116	PMID: 8657588
		AGC TGT GAA GCG AAG AGA CC	559		Reverse primer	---	Niittynen M, 2008[1]

[1]Niittynena M., Tuomistoa J.T., Pohjanvirtabc R.; Effect of 2,3,7,8-tetrachlorodibenzo-p-dioxin (TCDD) on heme oxygenase-1, biliverdin IXα reductase and δ-aminolevulinic acid synthetase 1 in rats with wild-type or variant AH receptor. Toxicol. 2008 ,250 , 132-142, doi: 10.1016/j.tox.2008.06.014

Table S5: Optimised protocol and validation studies for newly established HO-2 and BVRA primer pair using cDNA pool dilution series.

Target	Annealing temp (°C)/time (s)	Extension temp (°C)/time (s)	ΔCq (RT+ to NRT)[1]	slope	Correlation Coefficient (Pearson) R^2	Verified dynamic range
**HO-2**	62/30	72/40	18.06	-3.275	1.000	4^5
**BVRA**	62/30	72/40	12.00	-3.321	0.997	4^5

[1] Least difference detected between cDNA (RT+) and respective no reverse transcription control (NRT) in all samples analyzed.

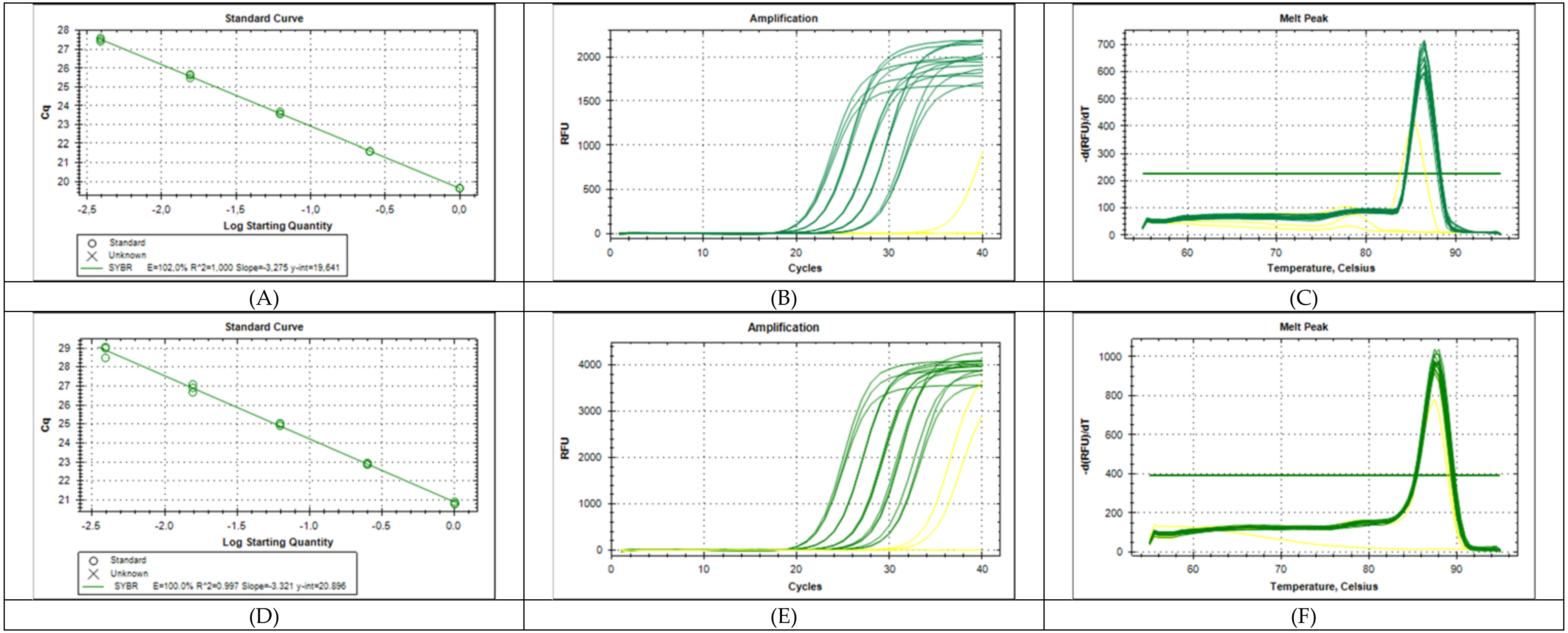

Figure S2: Validation of the HO-2 and BVRA primer pair. The suitability of the newly established HO-2 (A-C) and BVRA qPCR assays (D-F). Primer pairs were validated in separate experiments by performing qPCR measurements of (A, D) standard curves prepared from a cDNA pool. In (B, E) amplification plots and (C, F) melt curve analyses samples are shown in green, while no reverse transcription controls (NRT) are shown in yellow.
